# Mesophilic Argonaute-based isothermal detection of SARS-CoV-2

**DOI:** 10.3389/fmicb.2022.957977

**Published:** 2022-07-27

**Authors:** Xiao Li, Huarong Dong, Xiang Guo, Fei Huang, Xiaoyi Xu, Nuolan Li, Yu Yang, Tianbao Yao, Yan Feng, Qian Liu

**Affiliations:** ^1^State Key Laboratory of Microbial Metabolism, School of Life Sciences and Biotechnology, Shanghai Jiao Tong University, Shanghai, China; ^2^Department of Cardiology, Ren Ji Hospital, Shanghai Jiao Tong University School of Medicine, Shanghai, China

**Keywords:** mesophilic Ago, RNA viruses, isothermal detection, one-step method, SARS-CoV-2

## Abstract

Coronavirus disease (COVID-19), caused by SARS-CoV-2 infection and its mutations, has spread rapidly all over the world and still requires sensitive detection to distinguish mutations. CRISPR-based diagnosis has been regarded as a next-generation detection method; however, it has some limitations, such as the need for specific recognition sequences and multiple enzymes for multiplex detection. Therefore, research on the exploration and development of novel nucleases helps to promote specific and sensitive diagnoses. Prokaryotic Argonaute (Ago) proteins exert directed nuclease activity that can target any sequence. Recently, thermophilic Agos have been developed as new detection techniques achieving multiplexity for multiple targets using a single enzyme, as well as accurate recognition of single-base differential sequences. In this study, to overcome the requirement for high reaction temperature of thermophilic Ago-based methods, we expanded the mining of mesophilic Agos to achieve CRISPR-like isothermal detection, named mesophilic Ago-based isothermal detection method (MAIDEN). The principle of MAIDEN uses mesophilic Ago cleavage combined with reverse transcription, which can provide single-strand DNA as a substrate and allow cleavage of fluorescence probes to sense SARS-CoV-2 at moderate temperature. We first mined and optimized the mesophilic Ago and the fluorescence reporter system and then selected a compatible reverse transcription reaction. Furthermore, we optimized MAIDEN into a one-step reaction that can detect SARS-CoV-2 RNA at the nanomolar concentration at a constant temperature of 42°C within 60 min. Therefore, MAIDEN shows advantageous portability and easy-to-implement operation, avoiding the possibility of open-lid contamination. Our study was the first attempt to demonstrate that mesophilic Agos can be harnessed as diagnostic tools, and MAIDEN was easily extended to detect other pathogens in a rapid and efficient manner.

## Introduction

Since 2020, coronavirus disease (COVID-19), caused by SARS-CoV-2, has spread rapidly across the globe, seriously affecting public health and economic development (Dhama et al., 2020; Standl et al., 2021). SARS-CoV-2 is an RNA coronavirus, which exhibits high-frequency gene mutation that is closely related to virus virulence and transmissibility (Korber et al., 2020; Ali et al., 2021; Wang et al., 2021b). Due to its rapid infectivity, developing a nucleic acid method that accurately identifies virus variants for point-of-care testing (POCT) is of great importance. Currently, nucleic acid detection based on qRT-PCR is the gold standard for SARS-CoV-2 diagnosis (Oliveira et al., 2020); however, the requirements for specialized instruments and technicians limit its application in POCT diagnosis (Cevik et al., 2020).

The discovery and application of programmable nucleases have revolutionized their use in the field of diagnostics with improved sensitivity and feasible on-site analysis (Chandrasegaran and Carroll, 2016; Batista and Pacheco, 2018). In particular, the recently reported diagnostic technology based on the CRISPR/Cas system is acknowledged as one of the “seven technologies to watch in 2022” (Eisenstein, 2022). CRISPR-based diagnostics mainly rely on PAM-dependent cleavage of Cas9 or collateral cleavage of Cas12/13/14 to generate a detectable fluorescence signal (Gootenberg et al., 2017; Harrington et al., 2018; Li et al., 2018; Chen et al., 2021). Consequently, the target nucleic acid leaves a strong signature that allows detection through a fluorescence instrument or paper test. For example, several reports have demonstrated a rapid, portable CRISPR-based test for SARS-CoV-2 detection (Aman et al., 2020; Broughton et al., 2020; Ding et al., 2020; Liu et al., 2021b). According to these reports, there are still some limitations, such as PAM/PFS sequence restriction of the target sequence, potential degradation and high cost of long RNA guides, and multiplex detection requiring multiple Cas enzymes (Li et al., 2019). Therefore, there has been much interest in the mining of novel nucleases for the specific detection of target nucleic acids.

The prokaryotic Argonaute (Ago) protein shows nuclease activity for target nucleic acids directed by short nucleic acids as a guide DNA (gDNA; Hegge et al., 2018). Benefiting from its cleavage activity does not require specific recognition sequences, Ago can target arbitrary sequences with gDNAs that are easy to design and synthesize at a low cost (Swarts et al., 2015; Enghiad and Zhao, 2017). In recent years, thermophilic Agos have stimulated the development of novel detection techniques (Hegge et al., 2019; Song et al., 2020; Brandolini et al., 2021; Liu et al., 2021a; Wang et al., 2021a; Xun et al., 2021a,b; Qin et al., 2022; Ye et al., 2022). Compared with CRISPR-Cas enzymes, thermophilic Agos present distinct advantages in accurate recognition of single-nucleotide differential sequences and the orthogonal capability of a single enzyme to achieve multiplex detection for multiple targets (Wang et al., 2021a; Xun et al., 2021b; Ye et al., 2022). Nevertheless, the existing detection methods based on thermophilic Agos still require heating equipment to maintain Ago activity, which restricts POCT application (Qin et al., 2022). To support a more portable solution, mesophilic Agos that can cleave targets at a normal temperature would provide a promising option to develop CRISPR-like isothermal detection. As far as we know, mesophilic Agos have been biochemistry characterized since 2019 (Hegge et al., 2019; Kuzmenko et al., 2019; Liu et al., 2021c), but their application for gene detection still requires further investigation.

To address the shortcomings of CRISPR and thermophilic Agos, we designed a one-step mesophilic Ago-based isothermal detection method (MAIDEN) based on the systematic study of mesophilic Agos and their compatibility with reverse transcription, which can provide a single-stranded DNA (ssDNA) target for optimal Ago cleavage of a fluorescent signal at moderate temperature for SARS-CoV-2 detection. On this basis, our study is the first to demonstrate that mesophilic Agos can be used as a promising diagnostic tool. As they exhibit specific cleavage that distinguishes targets with single-nucleotide differences, further use of mesophilic Agos is expected in the identification of SARS-CoV-2 and its mutants and can be easily extended to POCT applications in the future.

## Materials and methods

### Protein purification and activity assay

Mesophilic Ago proteins were expressed and purified as previously described by Dong et al. (2021) and Liu et al. (2021c). The DNA-guided DNA-cleaving activity was then compared, and the cleavage system was as described by Dong et al. (2021).

To verify the mesophilic Ago stepwise cleavage ability, a 10 μl system containing 1 × reaction buffer [containing 15 mM Tris-HCl (pH 8.0), 2 M NaCl], 2 mM MnCl_2_, 6 μM Ago, 0.1 μM 76 nt ssDNA, 0.25 μM of each primary gDNA, and 0.2 μM fluorescent reporter was mixed and centrifuged, and the reaction was carried out at 37°C for 60 min. Finally, 10 μl of 2 × miRNA deionized formamide gel loading buffer was added to terminate the reaction. The cleavage products were analyzed *via* electrophoresis with 16% TBE-urea PAGE gel and stained with GelRed nucleic acid dye, and the results were observed using a gel imaging system (Tanon, Shanghai, China). The nucleic acid sequences are summarized in Supplementary Table 1. All the used oligos were synthesized by Sangon Biotech (Shanghai, China); 2 × miRNA deionized formamide gel loading buffer was obtained from Sangon Biotech (Shanghai, China).

### RNA template preparation

To obtain the RNA target for the assay reaction, first, a T7 RNA polymerase recognition sequence and a gene fragment of the SARS-CoV-2 *1b RdRP* gene of about 200 bp were synthesized into the pUC19 plasmid. Then, amplification and preservation were performed through plasmid transformation, followed by amplification of the corresponding target fragment through PCR, and finally, transcription using a HiScribe T7 Quick High Yield RNA Synthesis Kit (New England Biolabs, MA, United States) to obtain the target RNA template fragment. The amplification region sequence of the *RdRP* gene of SARS-CoV-2 and the PCR primer sequences are summarized in Supplementary Table 1. All plasmids and primers were synthesized by Sangon Biotech (Shanghai, China).

### Reverse transcription and RNase H digestion

The sequences of the reverse-transcribed region of the *RdRp* gene of SARS-CoV-2 and the reverse transcription primer sequences are summarized in Supplementary Table 1.

Briefly, 20 μl of reverse transcription reaction solution contained 0.5 mM deoxynucleotides (Sangon Biotech, Shanghai, China), 1 × ProtoScript^®^ II reverse transcriptase reaction buffer (New England Biolabs, MA, United States), 0.2 μM reverse transcription primer, 10 mM DTT, 8 units of RNase inhibitor (New England Biolabs, MA, United States), 200 units of ProtoScript^®^ II reverse transcriptase (New England Biolabs, MA, United States), and 1 μl of RNA sample or nuclease-free water (as a negative control). Reverse transcription reactions were performed at 42°C for 1 h. To the above reaction system, 5 units of RNase H (New England Biolabs, MA, United States) was added and incubated at 37°C for 20 min to degrade the RNA in the DNA-RNA hybrid strand.

### Mesophilic Ago-mediated fluorescence detection

The sequences of the fluorescent reporter and gDNAs that recognize specific complementary DNA (cDNA) sequences are shown in Supplementary Table 1. For the purpose of fluorescence detection, the stepwise cleavage reaction of Ago was performed at 37°C for 1 h. To the above reaction system, a final concentration of 0.267 μM ssDNA reporter, MgCl_2_, primary gDNAs, and Ago were added with nuclease-free water to make up a 30 μl system. In the case of the *Pb*Ago detection system, target DNA (tDNA), *Pb*Ago, and gDNA were diluted at a final concentration ratio of 40:324:5, respectively, and the final concentration of MgCl_2_ was 0.33 mM. In the case of the *Km*Ago detection system, tDNA, *Km*Ago, and gDNA were diluted at ration of 40:540:1, respectively, and MgCl_2_ concentration was 1.00 mM. The system was placed on a fluorescence PCR instrument for detection (the fluorescence signal was detected every minute).

### One-step method for SARS-CoV-2 detection

The reverse transcription reaction reagents and the Ago stepwise cleavage reaction solution were added simultaneously. Briefly, the 30 μl reaction system contained 0.33 mM deoxynucleotides, ProtoScript^®^ II reverse transcriptase reaction buffer, 0.13 μM reverse transcription primer, 6.67 mM DTT, 8 units of RNase inhibitor, 200 units of ProtoScript^®^ II reverse transcriptase, 0.267 μM ssDNA reporter, MgCl_2_, primary gDNAs, mesophilic Ago, and 1 μl of RNA sample or nuclease-free water. The concentration conditions of tDNA, Ago, gDNA, and MgCl_2_ are the same as those described in the fluorescence detection reaction in the “Mesophilic Ago-mediated fluorescence detection” section. One-step reactions were performed in qPCR at 42°C for 1 h, with fluorescence collected every minute.

### Statistical analysis

All experimental results were shown as mean ± SD unless stated otherwise. Statistical significance for comparisons of more than two groups was determined using one-way ANOVA. Significance was considered as ^*^p < 0.05, ^**^p < 0.01, and ^***^p < 0.001. Statistical analyses were carried out with GraphPad Prism 8.0.2.

## Results

### Principles and operation of reverse transcription and mesophilic Ago-mediated SARS-CoV-2 detection

**Figure 1:**
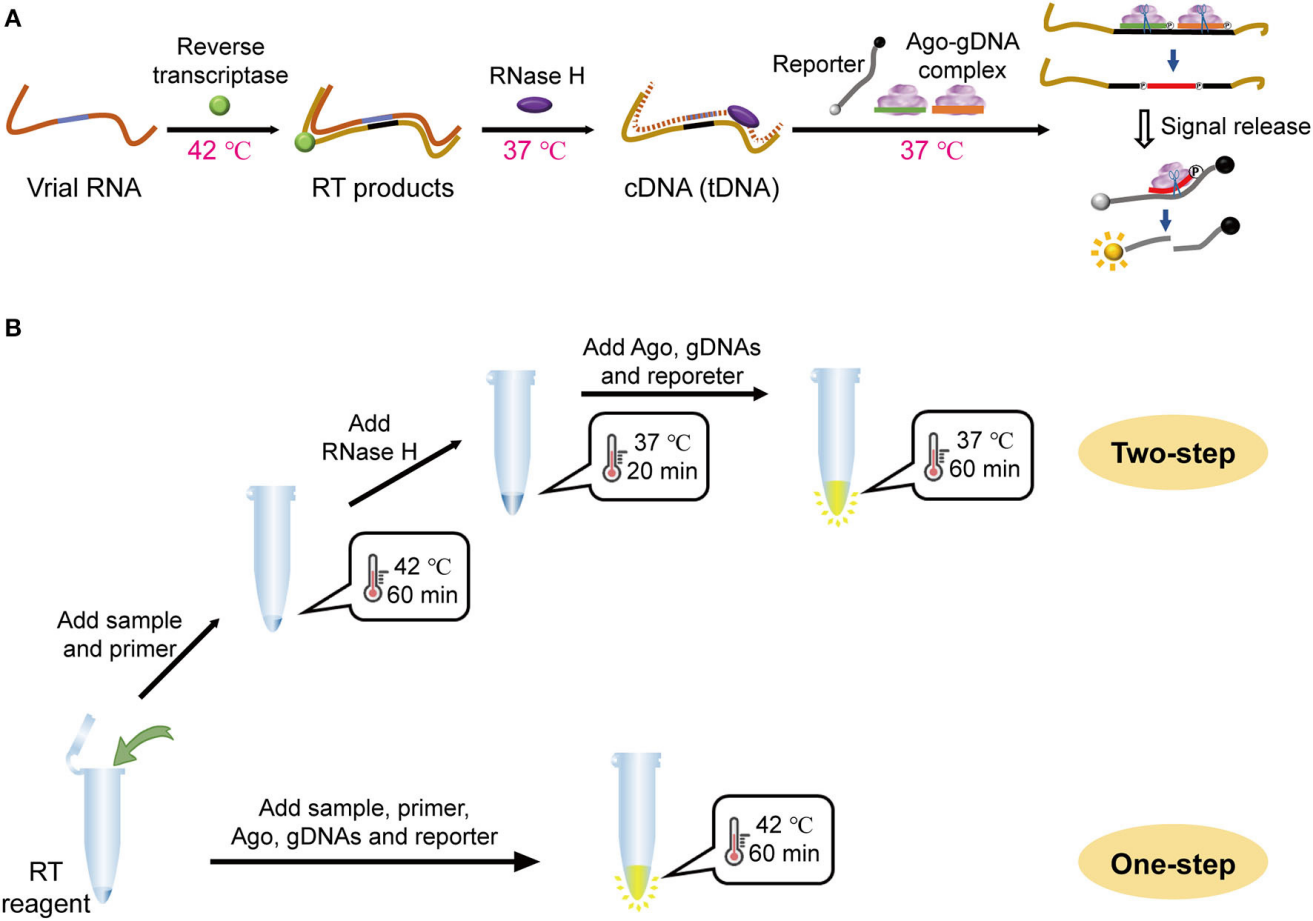
Workflow of the MAIDEN system. **(A)** Schematic illustration of the enzymatic process of mesophilic Ago-based diagnostics for RNA viruses. **(B)** Schematic diagram of the procedure for the two-step operation and the one-step operation.

The MAIDEN principle and operation that incorporate mesophilic Ago-mediated detection with reverse transcription are shown in Figure 1A. Briefly, specific sequences of the *orf1a/b* gene from SARS-CoV-2 (Supplementary Table 1) were reverse transcribed using reverse transcriptase. RNA in the DNA-RNA hybrid strand resulting from reverse transcription was hydrolyzed using RNase H. Then, the target sequence of the resulting ssDNA could be recognized by a designed primary gDNA and interacted with the Ago-gDNA ribonucleoprotein complex. Ago proteins cleave target sequences separately under the specific guidance of the primary gDNAs, producing short ssDNAs as secondary gDNAs. Consequently, the Ago-secondary gDNA complex recognizes and cleaves the designed ssDNA reporter with fluorophores and quenchers labeled at both ends. Cleavage of the reporter allows fluorescence release. The combination of reverse transcription with Ago takes advantage of Ago sequence recognition and enzyme activity at moderate temperature, resulting in specific RNA detection.

In practice, it is technically feasible to perform both reverse transcription and mesophilic Ago secondary cleavage in a one-pot reaction, since the temperature ranges of the two reactions are similar. At the same time, it was found that neither the reverse transcription primer nor the RNA template had a secondary structure, and there was no need to specifically incorporate a high-low temperature denaturation process. Since the reverse transcriptase used is a recombinant M-MuLV reverse transcriptase with its own RNase H activity, further addition of RNase H and its digestion and denaturation steps could be eliminated. Based on the above analysis, we further optimized the two-step assay as a simplified single reaction by adding both a reverse transcription system and an Ago stepwise lysis system (Figure 1B). This assay is the first case using mesophilic Ago compatible with reverse transcription in a one-pot isothermal system to detect SARS-CoV-2.

### Mining for mesophilic Agos as detection tools

Since mesophilic Ago activity determines the detection limit, we screened and investigated for the optimal enzyme. We collected all reported mesophilic Agos and classified them into two groups, including the DNA-targeted Ago representative *Cb*Ago and universal Ago representative *Km*Ago (Hegge et al., 2019; Kuzmenko et al., 2019; Liu et al., 2021c). Then, we searched the two Ago sequences in the NCBI database, and 82 bacterial Agos were aligned as candidates to generate a phylogenetic tree (Supplementary Figure 1). We also analyzed for conserved DEDX motif as a catalytically active Ago. Finally, we selected four Ago candidates in each of the two groups, as highlighted in Supplementary Figure 1.

To identify the Ago with the highest activity and lowest binding affinity at 37°C, we purified and characterized all candidates; eight Agos, except *Bsf* Ago, showed high soluble expression (Supplementary Figure 2). For the group of DNA-targeted Agos, 76 nt ssDNA target and 19 nt 5′-P gDNA were used for cleavage experiments at 37°C. We found that *Bl*Ago and *Cb*Ago showed the highest cleavage activity, but the binding effect was also strong. Considering its moderate activity and weak binding affinity, *Pb*Ago was chosen as the tool of the detection system (Supplementary Figure 3). For the group of universal Agos, we used varying forms of identical DNA and RNA sequences as targets and guide strands. All four Agos displayed similarly universal cleavage activity to *Km*Ago. Since *Km*Ago and *Po*Ago showed stronger activity, and *Po*Ago showed lower expression, *Km*Ago was used for target cleavage in the detection systems (Supplementary Figure 4).

### Reporter system design based on stepwise cleavage activity of mesophilic Agos

**Figure 2:**
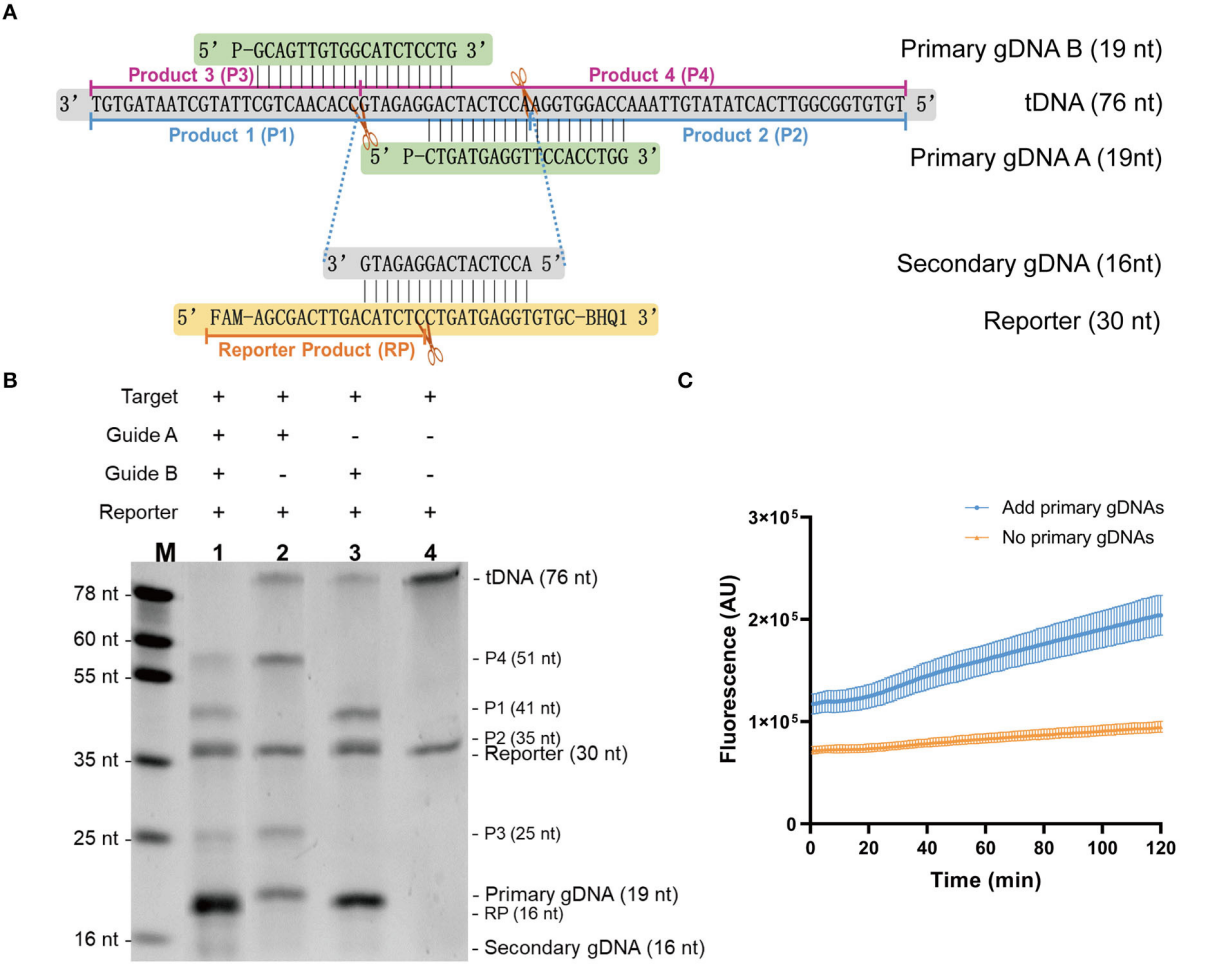
Validation of the stepwise cleavage activity of mesophilic Ago. **(A)** Schematic diagram of Ago stepwise cleavage. **(B)** Gel image of *Pb*Ago stepwise cleavage. **(C)** Fluorescent result of *Km*Ago stepwise cleavage. Data were collected from three independent experiments and are presented as the mean ± SD.

In this study, we aimed to establish an isothermal assay based on the stepwise cleavage activity of mesophilic Ago. To design a fluorescent reporter system, we investigated whether mesophilic Ago possesses this activity. Using SARS-CoV-2 *1b* ssDNA as target, we used *Pb*Ago as an example with a designed primary guide and fluorescent reporter at 37°C. Figure 2A shows a schematic and sequence diagram of Ago stepwise cleavage. The cleavage system was verified using electrophoresis (Figure 2B). We observed that *Pb*Ago cleaved the target ssDNA under the guidance of the primary gDNA and produced 16 nt of secondary gDNA, as expected. Then, the fluorescent reporter was further cleaved to yield the product. This process of fluorescent release was also monitored in real-time using *Km*Ago (Figure 2C). Collectively, it was determined that mesophilic Ago exerts stepwise cleavage activity and can act as a detection system.

### Screening enzymes for reverse transcription

As mesophilic Ago cleaves ssDNA as a substrate, we applied reverse transcription to transform the target RNA into a cleavage substrate. The efficiency of reverse transcriptase and the cleavage efficiency of Ago in this reaction buffer play a key role in this assay. Therefore, it is crucial to screen for an efficient reverse transcriptase as well as a suitable buffer compatible with the mesophilic cleavage reaction. We selected five reverse transcriptases and their commonly used buffers. As shown in Supplementary Figure 5A, four reverse transcriptases showed similarly stable reverse transcription efficiencies, except for reverse transcriptase No.3. After the reverse transcription reaction, we used RNase H to digest the RNA in the DNA-RNA hybrid strand and subsequently added *Pb*Ago and gDNA for the initial cleavage. As shown in Supplementary Figure 5B, a clear cleavage fragment was observed in the reaction containing reverse transcriptase No.5. Therefore, we established a reverse transcription reaction compatible with the subsequent mesophilic Ago cleavage.

### Optimization of an isothermal reaction of mesophilic Agos

**Figure 3:**
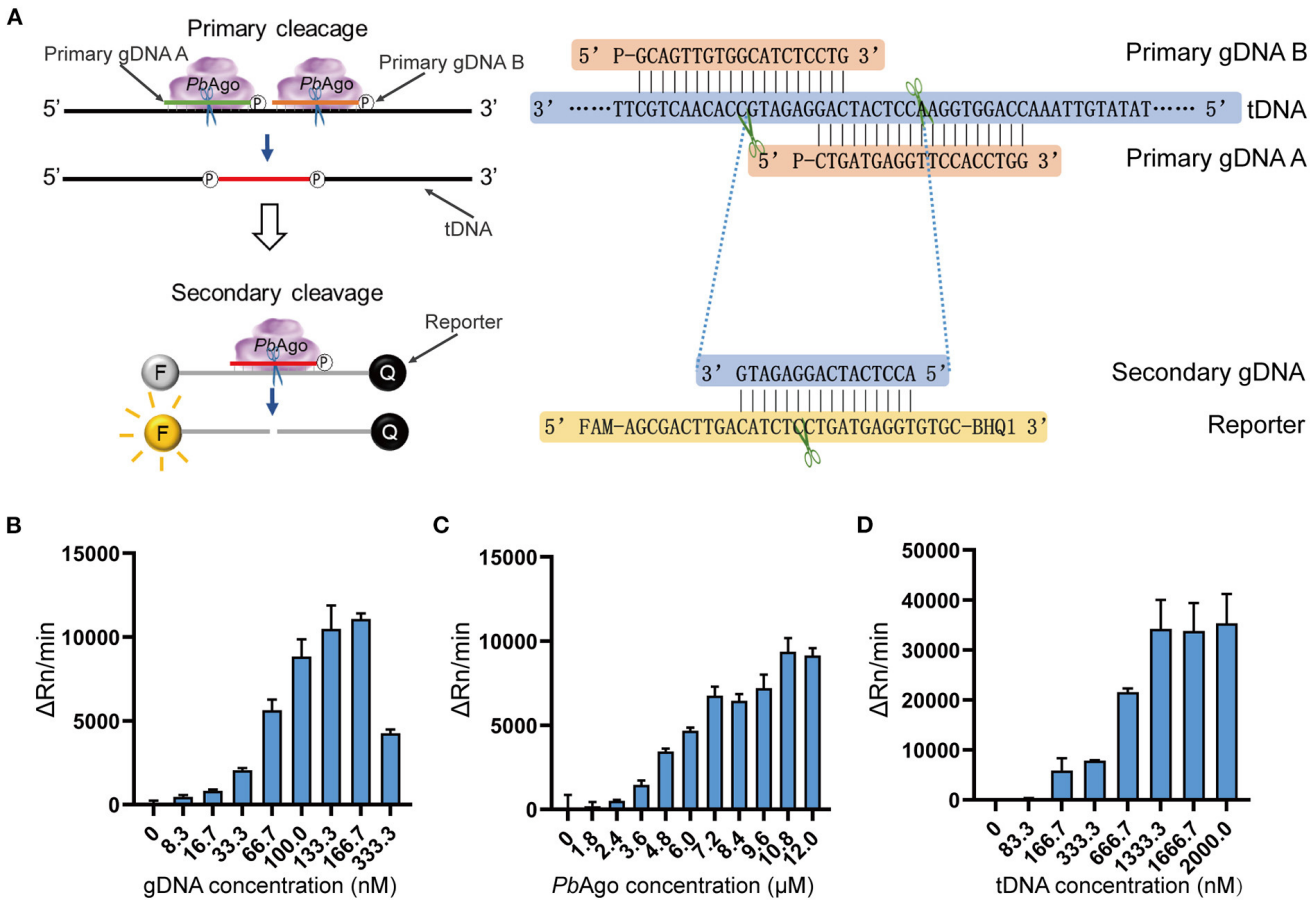
Optimization of an isothermal detection reaction of mesophilic Agos. **(A)** Schematic and sequence diagram of stepwise clevage in the isothermal detection system. **(B–D)** Fluorescence values of *Pb*Ago stepwise cleavage under different concentrations of gDNA **(B)**, *Pb*Ago **(C)**, and tDNA **(D)**.

To improve assay sensitivity, a series of optimizations were performed for the mesophilic Ago stepwise cleavage-mediated RNA detection system (Figure 3A). We evaluated the effect of different Mg^2+^ concentrations on the efficiency of the assay system. An additional 0.33 mM Mg^2+^ was added to the *Pb*Ago detection system (Supplementary Figure 6A), and an additional 1.00 mM Mg^2+^ was added to the *Km*Ago detection system (Supplementary Figure 6B). We also conducted concentration gradient experiments for Ago, gDNA, and tDNA in the system. The final molar concentration ratio of *Pb*Ago:gDNA:tDNA was confirmed to be 324:5:40 (Figures 3B–D), respectively. The final molar concentration ratio of *Km*Ago:gDNA:tDNA was 540:1:40 (Supplementary Figures 7A–C), respectively. Therefore, the above conditions were used for the reaction in the following assay system.

### Establishment and evaluation of mesophilic Ago-based detection method

The currently reported nucleic acid assays for SARS-CoV-2 mainly target its *E, N*, or *orf1a/b* genes. Therefore, we manually prepared the SARS-CoV-2 *1b* gene using the plasmid carrying 200 bp fragment of SARS-CoV-2 *1b* with a commercial RNA synthesis kit (Supplementary Figure 8). This RNA template was then subjected to a mesophilic Ago stepwise assay (Figure 1B). Thus, reverse transcription, RNase H digestion, and mesophilic Ago stepwise cleavage were performed sequentially. To overcome the problem of open-lib contamination that may lead to false-positive assays, we simplified the operation steps into a single reaction. By omitting the RNase H digestion step and adding the reverse transcription system and the Ago stepwise cleavage reaction system simultaneously at the same temperature, a one-step assay was established (Figure 1B). This assay allowed for the rapid detection of SARS-CoV-2 at a constant temperature of 42°C.

**Figure 4:**
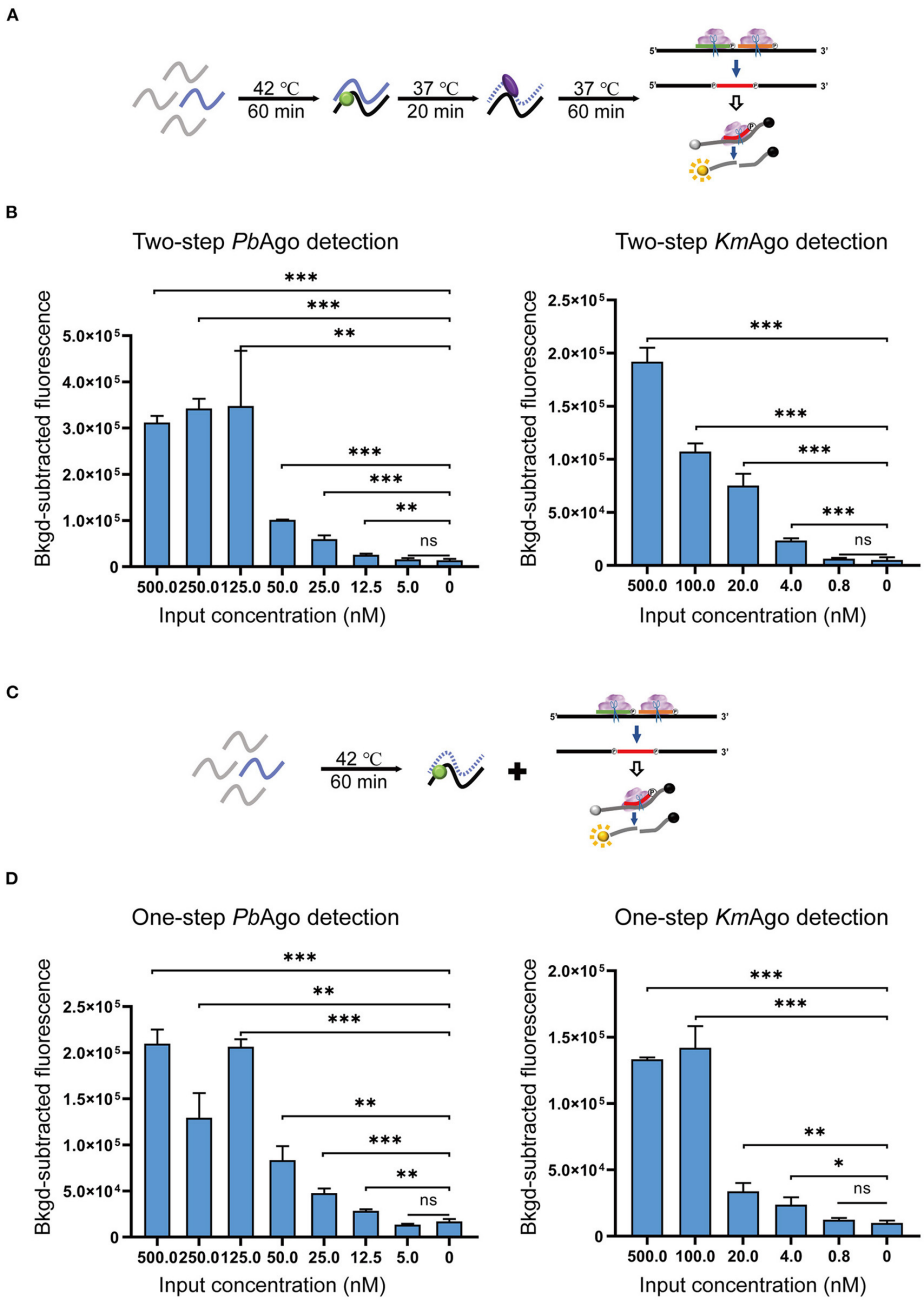
Sensitivity evaluation of the MAIDEN system. **(A)** Schematic diagram of the two-step operation. **(B)** Sensitivity of the *Pb*Ago and *Km*Ago-mediated two-step assay. **(C)** Schematic diagram of the one-step operation. **(D)** Sensitivity of the *Pb*Ago and *Km*Ago-mediated one-step assay. Data were collected from three independent experiments and are presented as the mean ± SD, and significance was considered as ns ≥ 0.05, *p < 0.05, **p < 0.01, and ***p < 0.001.

To evaluate the sensitivity of the mesophilic Ago isothermal method for SARS-CoV-2 detection, we diluted *in vitro* transcribed SARS-CoV-2 *1b* RNA in a gradient to nanomolar concentrations and assayed these diluted RNAs with optimized detection conditions to measure fluorescence in real time. Our results showed that two-step assays based on *Pb*Ago and *Km*Ago could detect viral RNA around 12.5 and 4.0 nM, respectively, and the modified one-step assays were also able to reach the same detection limits (Figures 4A–D). The results proved that Ago stepwise cleavage can compatible with reverse transcription process. We also suggested that a combination of additional isothermal amplification method was required to effectively detect lower concentrations of viral RNA.

## Discussion

In this study, we first established a one-pot method based on mesophilic Ago for simple, specific, and reliable detection of SARS-CoV-2 RNA. After systematically mining the mesophilic Agos, two *Km*Ago and *Pb*Ago candidates with optimal activity were chosen for the design of the fluorescence reporter system. Combined with the reverse transcription process, the ssDNA target could be served as a substrate for *Km*Ago and *Pb*Ago to generate a detectable fluorescence signal. Furthermore, the optimized one-pot assay could perform an operation without lid-opening to detect target RNA at the nM level in an hour. This method introduces mesophilic Agos to the diagnostic toolbox, presenting easy implementation and user-friendly POCT application and reducing contamination risk.

Programmable nucleic acid-binding proteins can target specific sequences and further cleave the target precisely, which would be a feasible tool to improve nucleic acid detection technology (Chandrasegaran and Carroll, 2016; Batista and Pacheco, 2018). In recent years, the gene-editing tool CRISPR has demonstrated applicability for viral diagnostics (Rahimi et al., 2021). Recently, CRISPR-based SARS-CoV-2 detection was accomplished with improved sensitivity and specificity (Supplementary Table 2). For instance, the CRISPR-FDS method developed by Huang et al. provided more reliable results than those reported in a clinical setting when the RT-qPCR assay was used (Huang et al., 2020; Rahimi et al., 2021). The STOPCovid method established by Zhang et al. is comparable in sensitivity to RT-qPCR-based techniques and is suitable for POCT diagnostic systems (Joung et al., 2020; Rahimi et al., 2021). However, CRISPR-based methods have some limitations as well. In Cas12b-based SARS-CoV-2 detection, a 111-nt guide RNA is required, leading to a risk of partial overlap between the guide RNA and one of the loop-mediated isothermal amplification (LAMP) primers (Broughton et al., 2020). Moreover, the target sequences are restricted to those with a PAM/PFS motif; for example, target N1 and N3 regions could not be designed due to the lack of suitable PAM sites for the Cas12 guide RNA (Broughton et al., 2020). Even though the one-pot reaction was achieved with decreased sensitivity, the amplification and Cas-based detection steps are operated separately in most CRISPR-based SARS-CoV-2 detection methods (Kellner et al., 2019). For easier use and reduced risk of contamination caused by lid-opening, attempts have been made to perform the reaction in closed cartridges, which has currently been accomplished using microfluidic reactors (Ramachandran et al., 2020; Rahimi et al., 2021).

The Ago system is generally involved in nucleic acid-guided host defense against invading nucleic acids in bacteria (Lisitskaya et al., 2018). Thermophilic Agos were first biochemically demonstrated to cleave ssDNA or RNA sequences that are complementary to the guide (Hegge et al., 2018). Owing to their programmable and specific nuclease nature similar to CRISPR, thermophilic Agos have been developed for applications in diagnosis. Thus far, several detection methods have been developed using thermophilic Agos (Supplementary Table 2). In particular, our group elucidated the stepwise activity of *Pf* Ago, which can uniquely exert double-stranded DNA (dsDNA) cleavage directed by a single gDNA rather than a pair of gDNAs that target each of the two strands (Xun et al., 2021a). Inspired by the stepwise *Pf* Ago activity, we then developed the renewed gDNA-assisted DNA cleavage with Argonaute (RADAR) method that demonstrated rapid and sensitive multiplex virus detection, enabling efficient genotype diagnosis at a single-nucleotide resolution (Xun et al., 2021a). The multiplex Ago-based nucleic acid detection system (MULAN) method integrated with LAMP and RADAR allowed simultaneous detection of SARS-CoV-2 and influenza viruses (Ye et al., 2022). Zhao et al. have developed *Pf* Ago-mediated nucleic acid detection (PAND) for SARS-CoV-2 detection based on a similar principle, which integrated *Pf* Ago cleavage and LAMP with prefabricated capillaries to enable a single-tube reaction (Xun et al., 2021b). Although these methods provide good sensitivity, they are limited by the need for a heating procedure and lack of compatibility with POCT application (Qin et al., 2022). As a consequence, mesophilic Agos as programmable nucleases at moderate reaction conditions have attracted great interest in nucleic acid detection.

This study successfully expanded mesophilic Ago applications in the diagnosis toolbox (Supplementary Table 2). First, mesophilic Agos were screened and evaluated, and *Km*Ago and *Pb*Ago were selected based on their higher cleavage activity, compared with other candidates. Similar to the reported thermophilic Agos, *Km*Ago, and *Pb*Ago exhibited stepwise activity, which is the basis for a fluorescent sensing system. Since mesophilic Agos only target single-strand nucleic acids as their substrate, and reverse transcription is a well-established process that produces ssDNA products from RNA viruses, we evaluated the compatibility of mesophilic Ago cleavage and reverse transcription. After screening for the optimal reverse transcription system, we optimized the reaction system components, including the concentration of divalent metal ions (Mg^2+^), primer selection, and Ago-to-guide ratio. Under optimal conditions, 60 min was sufficient for detection. In addition, we could even integrate the two separate reactions into a one-pot system without lid-opening, preventing contamination risk. Notably, the detection sensitivity of the one-pot reaction was similar to that of the two-step reaction, implying that mesophilic Agos can adapt to the selected reverse transcription reaction. Moreover, the one-pot reaction ensured completion within 1 h in a simple operation, exhibiting important application potential for on-site testing. The comparisons of our method with other reported CRISPR-based and thermophilic Ago-based methods for SARS-CoV-2 detection are listed in Supplementary Table 2.

In our previous study, *Pf* Ago was able to cleave the target sequence with a gDNA carrying a single-nucleotide mismatch (Liu et al., 2021a; Xun et al., 2021a; Ye et al., 2022). We then introduced an extra mismatch in the gDNA that yielded two contiguous mismatches to the target, which significantly decreased cleavage activity. Therefore, the MULAN method was developed to discriminate targets at single-base resolution, such as SARS-CoV-2 and its D614G mutant (Ye et al., 2022). Considering that both *Km*Ago and *Pb*Ago can distinguish targets with mismatches at certain positions (Dong et al., 2021; Liu et al., 2021c), we could introduce a mismatch in the reporter that resulted in a single-nucleotide mismatch with the target DNA, but two consecutive mismatches with the mutant DNA. This design enables the discrimination between wild-type and mutant in a single reaction. Consequently, we proposed that mesophilic Agos are also a powerful discrimination tool for emerging viral mutations.

The mesophilic Ago-based assay in this study is an encouraging attempt to detect SARS-CoV-2 RNA; however, the detection sensitivity can be further improved, compared with reported methods. We think that the selected mesophilic Agos used in combination with reverse transcriptase, which indicates an amplification-free process, may limit detection sensitivity. We propose a further combination with isothermal amplification methods, such as reverse transcription-recombinase aided amplification (RT-RAA), that would efficiently detect targets at a lower concentration. In addition, the cleavage activity of mesophilic Ago is much lower than that of thermophilic Ago that we previously used. We reasoned that the high temperature accelerates the enzymatic rate from a thermodynamic aspect. Therefore, we are planning to apply a protein engineering strategy to generate an evolved Ago with higher activity at moderate temperature, in order to improve the sensitivity of mesophilic Ago-based detection.

## Data availability statement

The raw data supporting the conclusions of this article will be made available by the authors, without undue reservation.

## Author contributions

XL, QL, and YF designed the study. XL, TY, and QL wrote the manuscript. XL, HD, NL, and YY performed the experiments and analyzed the data. XG, FH, and XX reviewed the manuscript. All authors contributed to the article and approved the submitted version.

## Funding

This study was supported by the National Key Research and Development Program of China (Grant No. 2018YFA0900403), the Shanghai Pilot Program for Basic Research—Shanghai Jiao Tong University (Grant No. 21TQ1400204) and the Natural Science Foundation of Shanghai (Grant No. 19ZR1430300).

## Conflict of interest

Shanghai Jiao Tong University has applied for a patent (application no. CN114277109A) for MAIDEN with QL, XL, YF, XG, and HD listed as co-inventors. The remaining authors declare that the research was conducted in the absence of any commercial or financial relationships that could be construed as a potential conflict of interest.

## Publisher's note

All claims expressed in this article are solely those of the authors and do not necessarily represent those of their affiliated organizations, or those of the publisher, the editors and the reviewers. Any product that may be evaluated in this article, or claim that may be made by its manufacturer, is not guaranteed or endorsed by the publisher.
