# Supplementary material for: Mesophilic Argonaute-based isothermal detection of SARS-CoV-2
Source: Front Microbiol. 2022 Jul 27;13:957977. doi: 10.3389/fmicb.2022.957977 (PMC9363790; doi:10.3389/fmicb.2022.957977)
Supplement: Supplementary file 1 [file Data_Sheet_1.pdf]

Supplementary Material

Supplementary Tables

Supplementary Table 1 The nucleotide sequences used in this research.

Single-stranded nucleic acids	Sequences（5’-3’）
SARS-CoV-2 1b 76 nt ssDNA	TGTGTGGCGGTTCACTATATGTTAAACCAGGTGGAACCTCATCAGGAGATGCCACAACTGCTTATGCTAATAGTGT
primary gDNA RT Guide A	P-GCAGTTGTGGCATCTCCTG
primary gDNA RT Guide B	P-CTGATGAGGTTCCACCTGG
Fluorescent Reporter	FAM-GCGACTTGACATCTCCTGATGAGGTGTGC-BHQ1
ssDNA target	ATATACTATACAACCTACTACCTCGTATAAATTTTTAAATAAATA-FAM
ssRNA target	AUAUACUAUACAACCUACUACCUCGUAUAAAUUUUUAAAUAAAUA-FAM
5’P DNA guide	P-TGAGGTAGTAGGTTGTATAGT
5’OH DNA guide	HO-TGAGGTAGTAGGTTGTATAGT
5’P RNA guide	P-UGAGGUAGUAGGUUGUAUAGU
5’OH RNA guide	HO-UGAGGUAGUAGGUUGUAUAGU
Inserted DNA fragment	TAATACGACTCACTATAGGGGATAAAAGTGCATTAACATTGGCCGTGACAGCTTGACAAATGTTAAAAACACTATTAGCATAAGCAGTTGTGGCATCTCCTGATGAGGTTCCACCTGGTTTAACATATAGTGAACCGCCACACATGACCATTTCACTCAATACTTGAGCACACTCATTAGCTAATCTATAGAAACGGTGTGACAAGCTACAACACGTTGT
FW-1b-256 bp	CAGCTATGACCATGATTACGCCAAG
RV-1b-256 bp	GGGGATCCACAACGTGTTGTAGCTT
RNA template	GGGGAUAAAAGUGCAUUAACAUUGGCCGUGACAGCUUGACAAAUGUUAAAAACACUAUUAGCAUAAGCAGUUGUGGCAUCUCCUGAUGAGGUUCCACCUGGUUUAACAUAUAGUGAACCGCCACACAUGACCAUUUCACUCAAUACUUGAGCACACUCAUUAGCUAAUCUAUAGAAACGGUGUGACAAGCUACAACACGUUGUGGAUCCCC
RT primer	ACAACGTGTTGTAGC

Supplementary Table 2 Comparison of reported CRISPR-based and thermophilic Ago-based methods for SARS-CoV-2 detection.

Detection methods	Nuclease	Operation	Target motif requirement	Guide oligos	Specificity	Lid opening	Portable
DETECTR (Broughton et al., 2020)	*Lb*Cas12a	62 °C for 20-30 min; 37 °C for 10 min	PAM	41 nt gRNAs	High	Yes	Yes
CRISPR-FDS (Huang et al., 2020)	*Lba*Cas12a	42 °C for 20 min; 37 °C for 20 min	PAM	41-43 nt gRNAs	Medium	Yes	No
CRISPR/Cas12a-NER (Wang et al., 2020)	*Fn*Cas12a	39 °C for30 min; 37 °C for 15 min	PAM	28 nt crRNAs	High	Yes	Yes
STOPCovid (Joung et al., 2020)	*Aap*Cas12b	60 °C for 1 h	PAM	111 nt crRNAs	High	No	Yes
CASdetec (Guo et al., 2020)	*Aa*Cas12b	42 °C for 30 min; 42 °C for 10-30 min	PAM	103-105 nt sgRNAs	High	No	Yes
SHERLOCK (Zhang et al., 2020)	*Lwa*Cas13a	42°C for 25 min; 37°C for 30 min	PFS	64 nt crRNAs	High	Yes	Yes
SARS-CoV-2 PAND (Wang et al., 2021)	*Pf*Ago	55 °C for 10 min; 45 PCR cycles; 95 °C for 20-30 min	No	16 nt gDNAs	High	Yes	No
MULAN (Ye et al., 2022)	*Pf*Ago	65 °C for 30-40 min; 95 °C for 10-15 min	No	16 nt gDNAs	High	No	Yes
MAIDEN	*Pb*Ago/*Km*Ago	42 °C for 1 h	No	19 nt gDNAs	High	No	Yes

Supplementary Figures

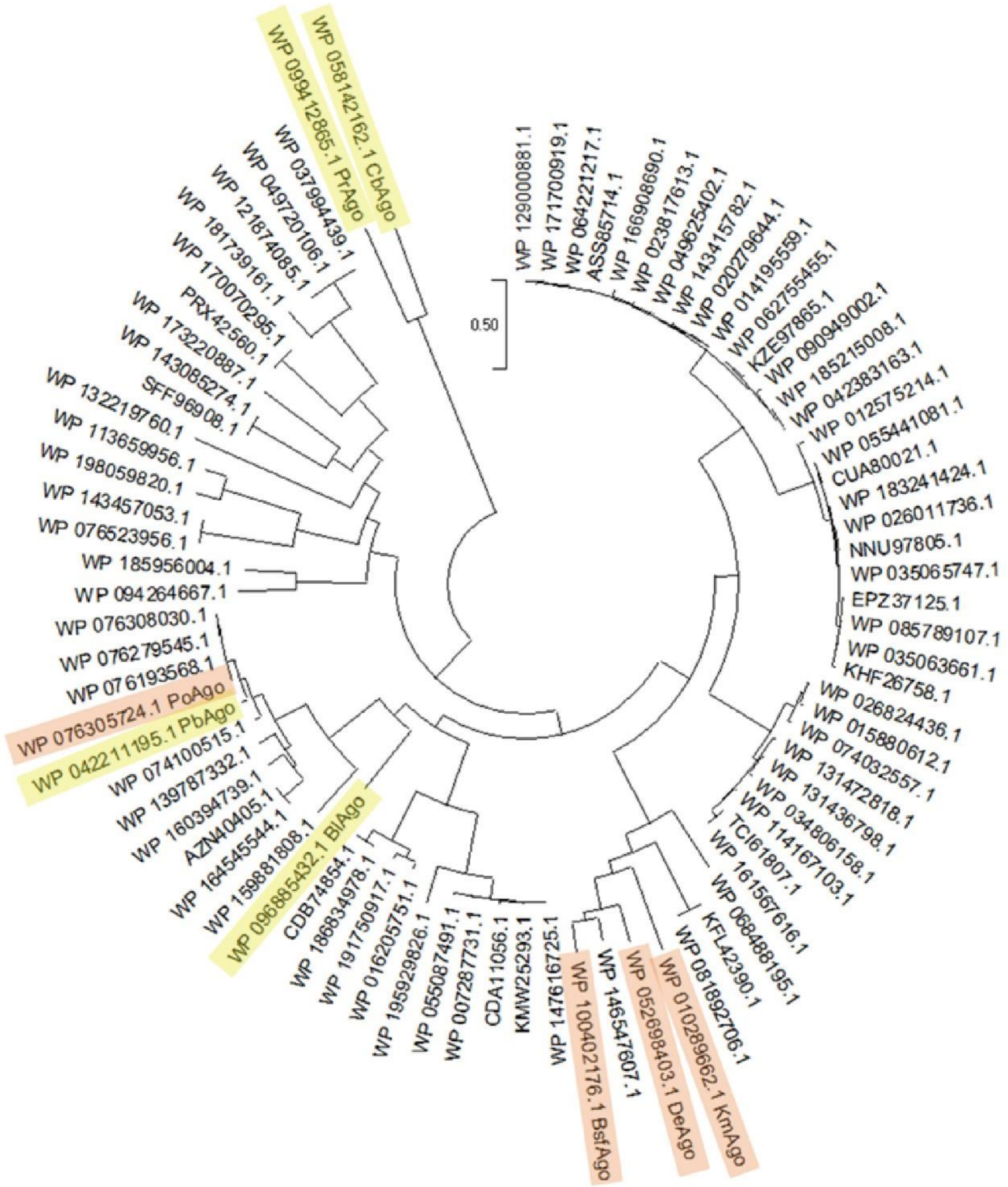

Supplementary Figure 1. Phylogenetic tree analysis based on the amino acid sequences of the characterized mesophilic Agos. Candidate Agos that target DNA are highlighted in yellow. Candidates for universal Ago are highlighted in orange. Ago from *Brevibacillus_laterosporus* (*Bl*Ago, WP_096885432.1); Ago from *Clostridium butyricum* (*Cb*Ago, WP_058142162.1); Ago from *Paenibacillus borealis* (*Pb*Ago, WP_042211195.1); Ago from *Pseudobutyrivibrio ruminis* (*Pr*Ago, WP_099412865.1); Ago from *Kurthia massiliensis* (*Km*Ago, WP_010289662.1); Ago from *Domibacillus enclensis* (*De*Ago, WP_052698403.1); Ago from *Bacillus sp. FJAT-42315* (*Bsf*Ago, WP_100402176.1); Ago from *Paenibacillus odorifer* (*Po*Ago, WP_076305724.1).

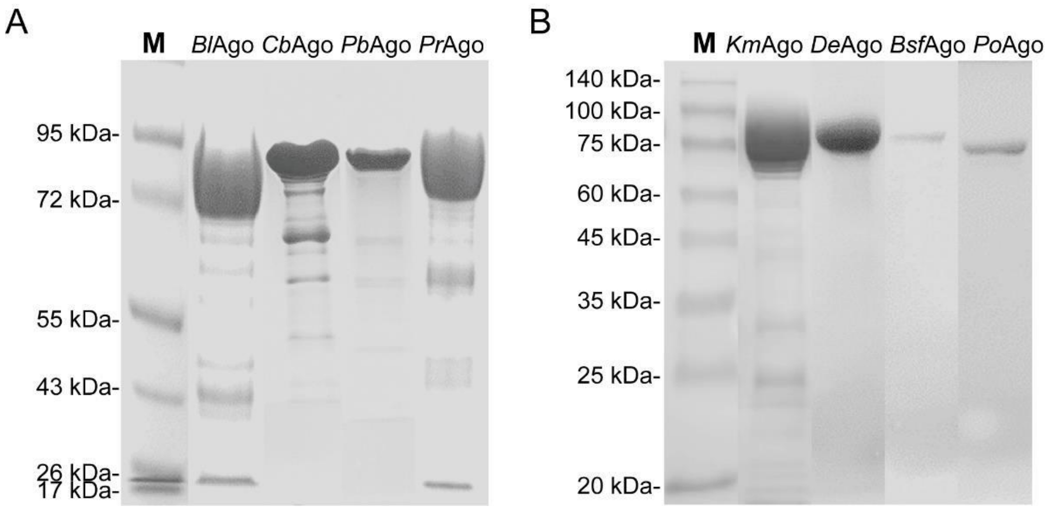

Supplementary Figure 2. SDS-PAGE analysis of purified Agos. (A) Candidate Agos that target DNA: *Bl*Ago, *Cb*Ago, *Pb*Ago, *Pr*Ago. (B) Candidates for universal Ago: *Km*Ago, *De*Ago, *Bsf*Ago, *Po*Ago.

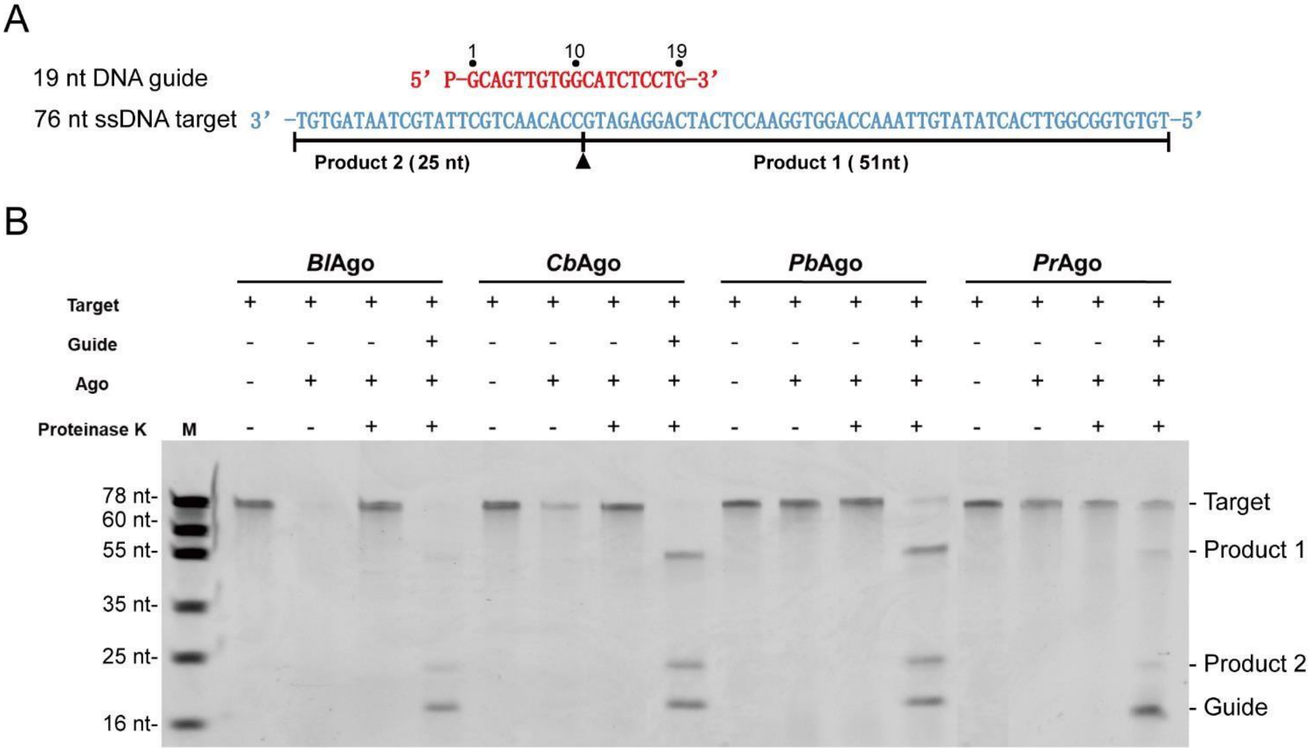

Supplementary Figure 3. *In vitro* cleavage activity determination of *Bl*Ago, *Cb*Ago, *Pb*Ago and *Pr*Ago. (A) Synthetic 5’P gDNA and ssDNA targets sequence. (B) The gel electrophoresis result of the Ago cleavage experiment.

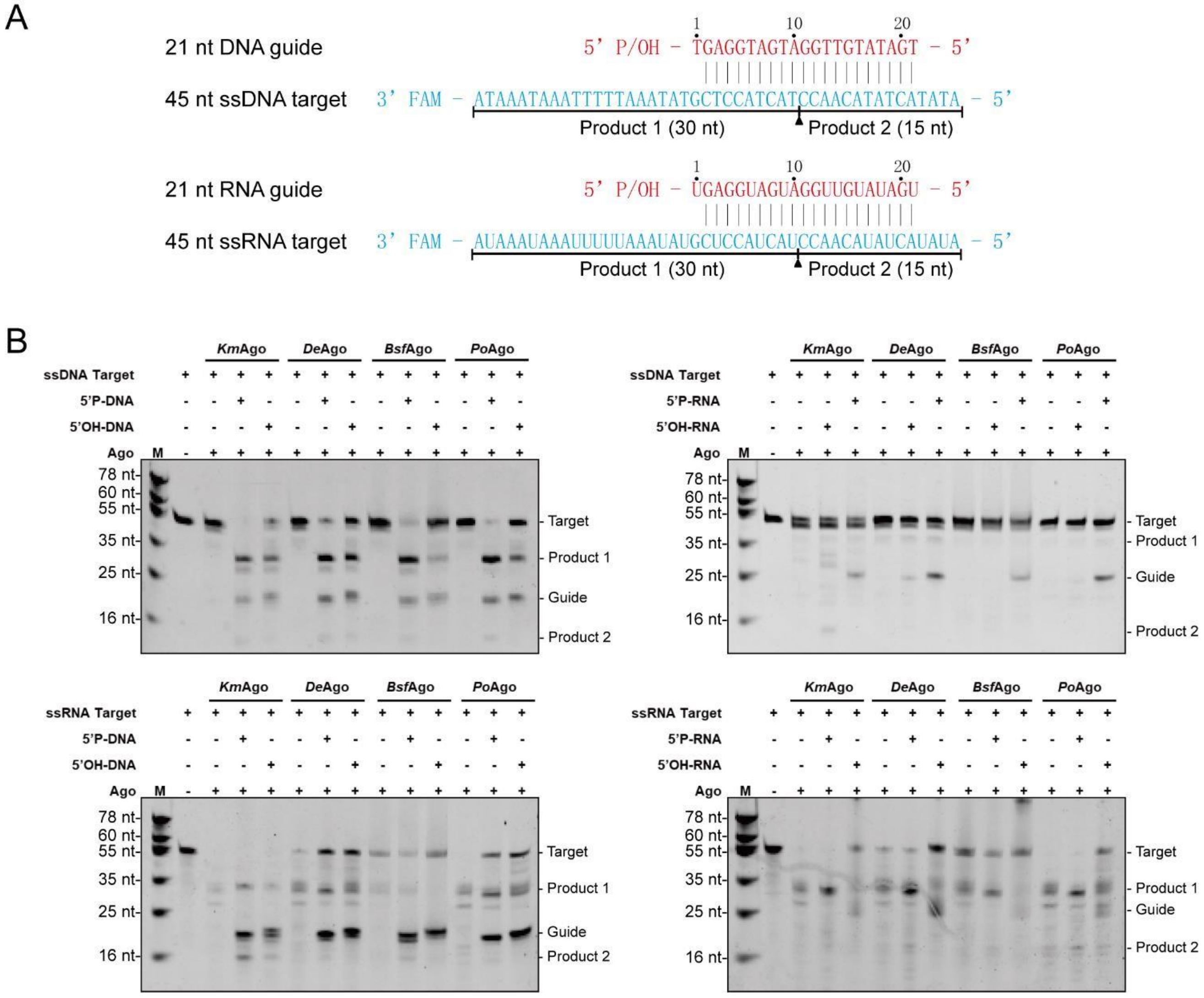

Supplementary Figure 4. *In vitro* cleavage activity determination of *Km*Ago, *De*Ago, *Bsf*Ago and *Po*Ago. (A) Synthetic 5’P and 5’OH gDNA, gRNA and ssDNA, RNA targets sequence. (B-E) The gel electrophoresis result of the Ago cleavage experiment: (B) DNA-guided cleavage of DNA target; (C) RNA-guided cleavage of DNA target; (D) DNA-guided cleavage of RNA target; (E) RNAguided cleavage of RNA target.

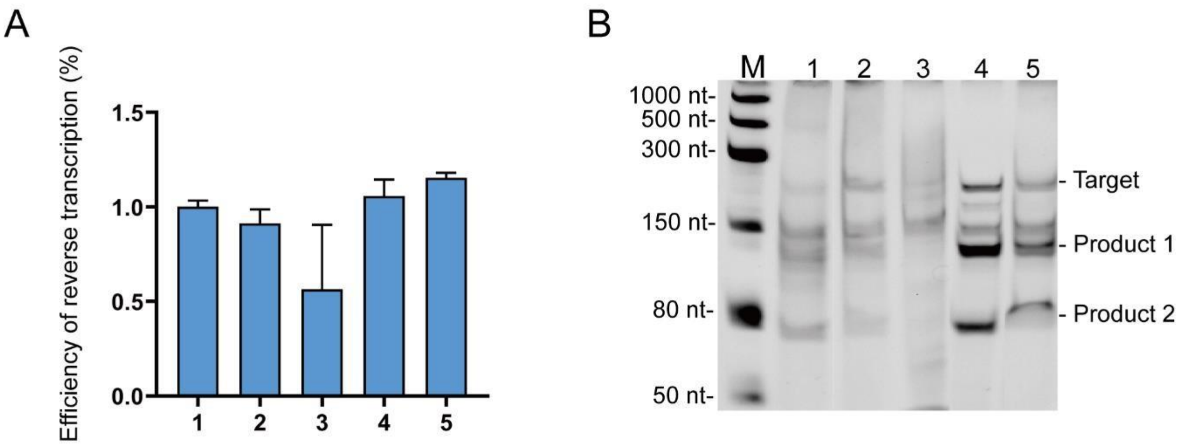

Supplementary Figure 5. Screening of enzymes for reverse transcription. (A) Transcription efficiency of the five reverse transcriptases. Error bars, standard deviation (SD) of replicates (n = 3). (B) Cleavage efficiency of *Pb*Ago in five reverse transcription systems.

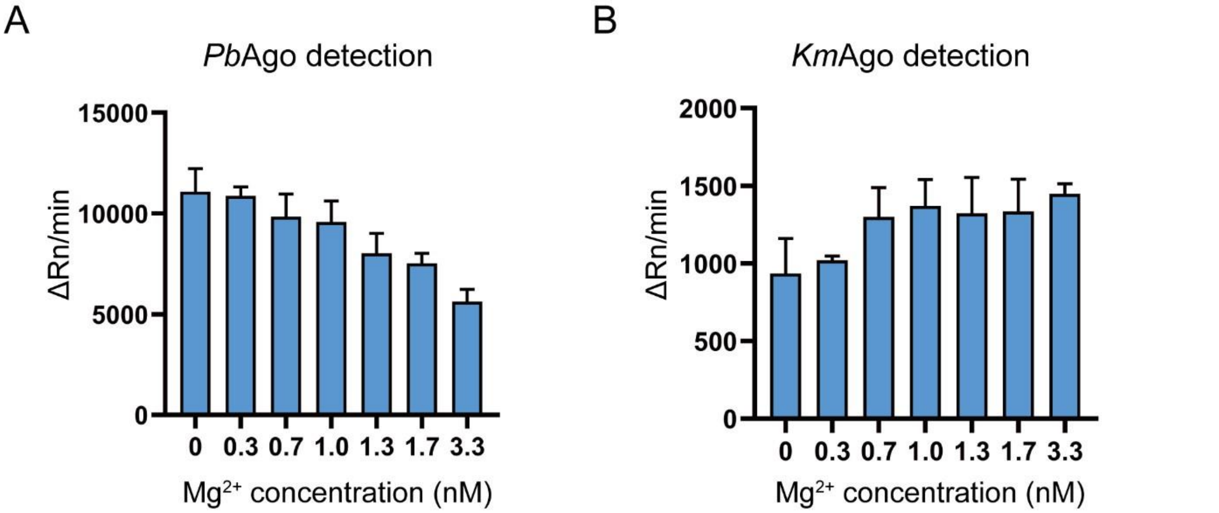

Supplementary Figure 6. Optimization of an isothermal detection reaction of mesophilic Agos. (A) Fluorescence values of *Pb*Ago cascade cleavage under different concentrations of Mg^{2+}. (B) Fluorescence values of *Km*Ago cascade cleavage under different concentrations of Mg^{2+}. Data were collected from three independent experiments and are presented as the Mean ± SD.

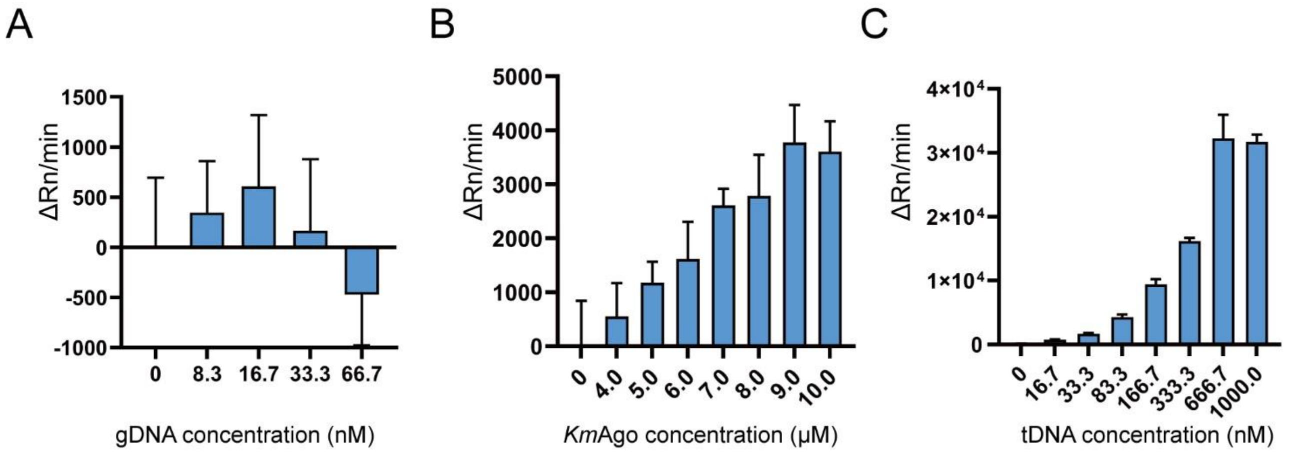

Supplementary Figure 7. Optimization of an isothermal detection reaction of *Km*Ago. (A-C) Fluorescence values of *Km*Ago cascade cleavage under different concentrations of gDNA (A), *Km*Ago (B), and tDNA (C). Data were collected from three independent experiments and are presented as the Mean ± SD.

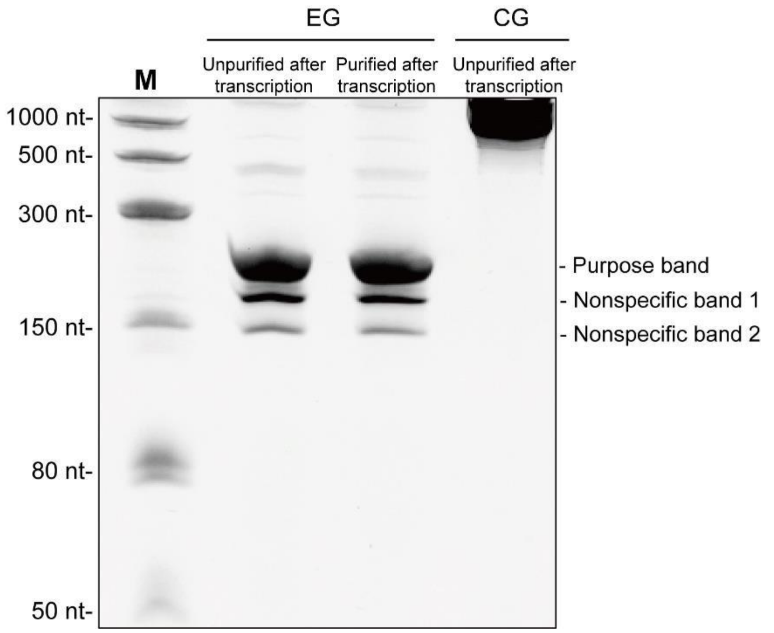

Supplementary Figure 8. The gel electrophoresis result of transcribed RNA. EG, experimental group, including unpurified and purified 200bp RNA template after transcription. CG, control group, unpurified RNA fragment transcribed from the DNA template that comes with the transcription kit.

Supplementary References

Broughton, J.P., Deng, X., Yu, G., Fasching, C.L., Servellita, V., Singh, J., et al. (2020). CRISPRCas12-based detection of SARS-CoV-2. *Nat. Biotechnol.* 38(7)**,** 870-874. doi: 10.1038/s41587020-0513-4.

Guo, L., Sun, X., Wang, X., Liang, C., Jiang, H., Gao, Q., et al. (2020). SARS-CoV-2 detection with CRISPR diagnostics. *Cell Discov.* 6, 34. doi: 10.1038/s41421-020-0174-y.

Huang, Z., Tian, D., Liu, Y., Lin, Z., Lyon, C.J., Lai, W., et al. (2020). Ultra-sensitive and highthroughput CRISPR-p owered COVID-19 diagnosis. *Biosens. Bioelectron.* 164**,** 112316. doi: 10.1016/j.bios.2020.112316.

Joung, J., Ladha, A., Saito, M., Segel, M., Bruneau, R., Huang, M.W., et al. (2020). Point-of-care testing for COVID-19 using SHERLOCK diagnostics. *medRxiv*. doi: 10.1101/2020.05.04.20091231.

Wang, F., Yang, J., He, R., Yu, X., Chen, S., Liu, Y., et al. (2021). PfAgo-based detection of SARSCoV-2. *Biosens. Bioelectron.* 177**,** 112932. doi: 10.1016/j.bios.2020.112932.

Wang, X., Zhong, M., Liu, Y., Ma, P., Dang, L., Meng, Q., et al. (2020). Rapid and sensitive detection of COVID-19 using CRISPR/Cas12a-based detection with naked eye readout, CRISPR/Cas12a-NER. *Sci. Bull. (Beijing)* 65(17)**,** 1436-1439. doi: 10.1016/j.scib.2020.04.041.

Ye, X., Zhou, H., Guo, X., Liu, D., Li, Z., Sun, J., et al. (2022). Argonaute-integrated isothermal amplification for rapid, portable, multiplex detection of SARS-CoV-2 and influenza viruses. *Biosens. Bioelectron.* 207, 114169. doi: 10.1016/j.bios.2022.114169.

Zhang, F., Abudayyeh, O.O., and Gootenberg, J.S. (2020). A protocol for detection of COVID-19 using CRISPR diagnostics. *Broad Institute* 8.
